# Assessing the levels of food shortage using the traffic light metaphor by analyzing the gathering and consumption of wild food plants, crop parts and crop residues in Konso, Ethiopia

**DOI:** 10.1186/1746-4269-8-30

**Published:** 2012-08-07

**Authors:** Dechassa Lemessa Ocho, Paul C Struik, Lisa L Price, Ensermu Kelbessa, Koshana Kolo

**Affiliations:** 1 UN-OCHA Ethiopia, P.O. Box 13158, Addis Ababa, Ethiopia; 2 Centre for Crop Systems Analysis, Wageningen University, P.O. Box 430, 6700, AK, Wageningen, The Netherlands; 3 Department of Anthropology, Oregon State University, 204 Waldo Hall, Corvallis, OR, 97331-6403, USA; 4 The National Herbarium, Biology Department, Science Faculty, Addis Ababa University, P.O. Box 3434, Addis Ababa, Ethiopia; 5 Parka Office, Karat Town, Konso District, Southern Nations and Nationalities and Peoples Regional Government, Ethiopia

**Keywords:** Crop residues, Ethiopia, Ethnobotany, Famine, Food situation assessment, Humanitarian relief, Indigenous knowledge, Konso, Traffic light metaphor, Wild food plants

## Abstract

**Background:**

Humanitarian relief agencies use scales to assess levels of critical food shortage to efficiently target and allocate food to the neediest. These scales are often labor-intensive. A lesser used approach is assessing gathering and consumption of wild food plants. This gathering per se is not a reliable signal of emerging food stress. However, the gathering and consumption of some specific plant species could be considered markers of food shortage, as it indicates that people are compelled to eat very poor or even health-threatening food.

**Methods:**

We used the traffic light metaphor to indicate normal (green), alarmingly low (amber) and fully depleted (red) food supplies and identified these conditions for Konso (Ethiopia) on the basis of wild food plants (WFPs), crop parts (crop parts not used for human consumption under normal conditions; CPs) and crop residues (CRs) being gathered and consumed. Plant specimens were collected for expert identification and deposition in the National Herbarium. Two hundred twenty individual households free-listed WFPs, CPs, and CRs gathered and consumed during times of food stress. Through focus group discussions, the species list from the free-listing that was further enriched through key informants interviews and own field observations was categorized into species used for green, amber and red conditions.

**Results:**

The study identified 113 WFPs (120 products/food items) whose gathering and consumption reflect the three traffic light metaphors: red, amber and green. We identified 25 food items for the red, 30 food items for the amber and 65 food items for the green metaphor. We also obtained reliable information on 21 different products/food items (from 17 crops) normally not consumed as food, reflecting the red or amber metaphor and 10 crop residues (from various crops), plus one recycled stuff which are used as emergency foods in the study area clearly indicating the severity of food stress (red metaphor) households are dealing with. Our traffic light metaphor proved useful to identify and closely monitor the types of WFPs, CPs, and CRs collected and consumed and their time of collection by subsistence households in rural settings. Examples of plant material only consumed under severe food stress included WFPs with health-threatening features like *Dobera glabra* (Forssk.) Juss. ex Poir. and *inkutayata*, parts of 17 crops with 21 food items conventionally not used as food (for example, maize tassels, husks, empty pods), ten crop residues (for example bran from various crops) and one recycled food item (*tata*).

**Conclusions:**

We have complemented the conventional seasonal food security assessment tool used by humanitarian partners by providing an easy, cheap tool to scale food stress encountered by subsistence farmers. In cognizance of environmental, socio-cultural differences in Ethiopia and other parts of the globe, we recommend analogous studies in other parts of Ethiopia and elsewhere in the world where recurrent food stress also occurs and where communities intensively use WFPs, CPs, and CRs to cope with food stress.

## Background

Humanitarian agencies delivering relief resources use combined intensity (severity) and magnitude (impact of the crisis) scales to assess famine and grade levels of critical food shortage. They do so in order to target and most efficiently allocate the ever-limited relief resources to the neediest. Very early assessment scales, designed to mitigate the crisis and prevent its deterioration, included the Indian Famine Codes devised in the 1880s and the Codes of Kenya developed for the northern region (Turkana communities), which coded three subsequent famine warnings or measurement levels of food insecurity: 1) near-scarcity, 2) scarcity, and 3) famine [1]. Since 2004, the two largest relief organizations, the World Food Program of the United Nations and the United States Agency for International Development (USAID), have adopted a six-level scale, using livelihoods’ measures and measurements of mortality and child malnutrition, to categorize intensity and magnitude of food insecurity: 1) food secure, 2) food insecure, 3) food crisis, 4) famine, 5) severe famine, and 6) extreme famine [2]. Furthermore, the Food and Agriculture Organization of the United Nations (FAO) in its Integrated Food Security and Humanitarian Phase Classification (IPC) identified and classified five phases [3]: 1) generally food secure, 2) chronically food insecure, 3) acute food and livelihood crisis, 4) humanitarian emergency, and 5) famine/humanitarian catastrophe. These scales are labor-intensive tools to be used at the grass-root and community level where in the field visual observations are made in these early-warning systems assessments of the degree of food stress.

Maxwell et al. [4] analyzed data from 14 surveys in crisis affected or chronically vulnerable and food insecure Sub-Saharan Africa countries, including Ethiopia. These surveys incorporated context-specific Coping Strategies Indices (CSI). These authors identified eleven coping behaviors of individual households that were common to all surveys and also appeared to follow a relatively similar order of frequency and severity. These eleven coping behaviors were: 1) limiting meal portion size, 2) reducing number of meals, 3) skipping entire days, 4) borrowing food, 5) eating less-preferred food, 6) buying food on credit, 7) eating wild foods, 8) eating seed stock, 9) sending household member to eat elsewhere, 10) sending member to beg, and 11) adults eating less [4]. Based on a re-analysis, using data from multiple countries, Maxwell et al. [4] reduced the number of most frequently mentioned behaviors to five: 1) reducing the number of meals, 2) limiting meal portion size, 3) eating less-preferred food, 4) prioritizing certain members of the household, and 5) borrowing food. This classification indicates severity of individual coping behaviors, but does not specifically include eating wild foods.

In Ethiopia, occurrence and availability of wild food plants (WFPs) strongly varies with the seasons: their abundance steadily increases during the rainy seasons but decreases in the dry seasons [5-8]. Gathering and consumption of WFPs take place both in normal times when food stock is enough and in times of food shortage but increase as household food stock decreases [5,6,8-10]. The collection and consumption of WFPs have been used as classical indicators of severe food stress in Congo [11,12] and in Ethiopia [6,13-15]. Corbett [16] reviewed major food crises in North Nigeria (1973/74), Wello (North Ethiopia) (1984/85) and Darfur (Sudan) (1984) and noted that in all cases, gathering wild foods was sequenced as the first stage of response employed by the victims.

Also in South Sudan, where war and drought have been recurrent and devastating during the past decades, a community-based food situation monitoring system observes the gathering of WFPs (including when gathering takes place, who is gathering, and at what intensity this occurs) to mark the severity of food stress in rural populations and to predict future food stress [17,18]. This monitoring system has been in effect in many counties of South Sudan since 1997 and successfully predicted the 1998 famine [17]. The system receives increasing recognition as a valid tool.

Gathering requires a level of indigenous knowledge on endemic WFPs [17]. This knowledge might be even more important when gathering is part of a strategy to cope with hunger [17,18]. In Bahr el Ghazal State of Southern Sudan different counties had different levels of mortality during the 1998 famine where thousands of people died due to hunger. To some extent, these differences might be related to the level of knowledge of indigenous food plants [17,18].

In the national annual food security situation assessments in Ethiopia, gathering and consumption of WFPs are used as indicator of critical food shortage in all chronically food insecure areas of Ethiopia [15,19]. However, most studies to date have not analyzed the specifics of individual species but only broadly mentioned gathering of WFPs as an indicator of food stress. In addition, none of the recently published ethnobotanical studies carried out in Ethiopia (e.g., [8,10,20-23]) comprehensively related the gathering and consumption of specific WFPs to the early warning for severe food stress. In fact, unpublished studies conducted in the country by Getachew [8] and Guinand and Dechassa [5,6] provided some list of such plants in their studies. Yet, semi-pastoralist people in Ethiopia can identify specific plant species that they consume during periods of food shortage [5,8,19]. This suggests that it is not the gathering and consumption of all wild food plants per se that could mark current or emerging food stress, but rather the gathering and consumption of specific WFPs. The move to consumption of unattractive WFPs reflects the shift in consumption behavior *of adults* under conditions of food stress. The gathering and consumption of a WFP species with unpleasant taste, small gathering volume, poor availability, rarity, remote occurrence, or only occurring on inaccessible sites, and also those which cause extreme effort in processing into “food” (example *Dobera glabra* and *qachama* species of *pakanna* in Konso) could have a signaling function as such WFPs are not gathered in normal times but only consumed in extreme emergency situations [5-8,17]. Moreover, the previous studies do not report on the consumption of crop parts (CPs; crop parts which are not used for human consumption under normal conditions, such as maize tassels or leaves of Irish potato), and crop residues (CRs) gathered and consumed during times of food stress, as indicator for food shortage.

We surmise that there will be many WFPs, CPs and CRs with such a signaling function. Recording information on (the dynamics of) their gathering could thus provide a better tool to assess the food stress situation than merely observing that people start to gather WFPs. In this study, we present the classification of WFPs, CPs, and CRs into categories and behavior of the local subsistence farmers in Konso with regard to changes in food stress and the intensity of their gathering of selected plant foods in different categories when a severe food stress/famine is gradually building up. For such a classification we use the traffic light metaphor.

### The traffic light metaphor

The traffic light metaphor we use for the region of Konso, Ethiopia is composed of the following:

Green light: a normal satisfactory situation, when food may not be plentiful but generally is available to the community in adequate quantity and quality (*Hira*).

Amber light: a problematic situation, when food reserves are alarmingly low (*Sharshara*).

Red light: a situation in which the food supply is depleted (*Lawota kokoka*) and which requires emergency measures.

These three situations follow in sequence and illustrate the gradual deterioration of household food security. The amber light condition provides an early warning, while the red light condition indicates crisis conditions.

### Study area

Konso is a district located 585 km south of Addis Ababa. It is specifically suited to the analysis of whether gathering and consuming of specific wild food plants serves as a signal for severe food stress or not due to its extensive ethnobotanical and ethnoecological diversity and its frequent and recurrent droughts resulting in food stresses (notably extended ones, for example from 1996-1999 when the Konso rural community heavily relied on WFPs gathering and consumption [5]). Konso has a total population of 234,987 [24] with 90% of the rural population being farmers and the district having a rate of illiteracy of 96% [25]. In the midlands and mountainous areas, farmers are relying on an extremely laborious type of terrace agriculture on poor, gravelly and stony soils with low external input and low organic matter and recurrent droughts. The problem of low fertility is strongly enhanced by continuous over-cultivation of the soils for crop production without fallowing due to increasing land shortage [26]. About 90% of the households cultivate less than 1 ha of land [25].

The district (with an area of 2974 km^2^ and an altitude range of 650 to 2650 m above sea level) has a bimodal type of rainfall with a 30 year (1971–2000) average rainfall of 609 mm per year [26]. But the rains have become increasingly inadequate, erratic and unreliable [25-27]. The main rainy season is called *Belg* (*Para/Eapelta*) which occurs from mid-February/March to May. It is the major season for major crop production (with a 70% share of the annual production). The second and short rainy season (*Meher/Hagaya*) occurs from August/September to mid-October and is less important for crop production. *Meher*, however, is crucial for supporting sorghum ratoon (re-growth from previous sorghum stock), after the first harvest in June, more as feed than as food, mainly in the midland areas. Temperature in the district has little variation over the year, with an annual average of 22.4°C. Konso district has increasingly faced recurrent drought and this resulted into frequent occurrence of food shortage [25-27]. Therefore the people have traditionally developed adaptation strategies, including cleverly designed storage facilities. The culture is also rich in indigenous knowledge on wild plants as an important resource to be tapped as part of that adaptation strategy.

In most years, all wealth groups in Konso, including the better-off, receive food aid which covers on average 15% of their annual food requirements [26]. In Konso, there are two hunger seasons as farmers do not produce enough food, even not in ordinary years. The first and worst hunger season is from mid-September to October/November as sorghum, being the main staple food crop of the area (97% of the households depend on sorghum [26]), is only ready for harvest in December/January. The second hunger season is in April/May and extends up to mid-June [25-27]. This one occurs when weeding takes place and annual leafy WFPs are more abundant due to the *Belg* rains [5,6,8]. Hence the second hunger season is less devastating than the first one.

## Materials and methods

All interviews (those with individual households and those with key informants) and focus group discussions were carried out in 2006, 2008 and 2009. All field work of this study was strictly carried out by the senior author himself supported by a local field assistant/translator from the community (who also shared an indigenous language with the senior author for clear communication). The field assistant was academically trained in agriculture and served as an agronomist within the local government office.

### Individual interviews

A group of illiterate informants (including 120 women and 100 men and all coming from different households), who all grew up in the area and still lived there, was randomly selected for the study from the households list-book provided by the community leaders. Based on the reconnaissance study made in the study area before the main field work, these two hundred twenty individuals were asked to mention (in Konso language) foodstuffs: WFPs, CPs, and CRs gathered and consumed, during times of food stress. Each informant was interviewed only once and bystanders were avoided during the individual interviews. We used the technique of free-listing [28]. Each informant was requested to orally free-list the stuffs by using the agreed Konso cover term/phrase *“Lata lawota bata dhamman”*, which means foodstuffs eaten in food stress/food shortage times*.* When they free-listed the names of the items, the terms were jotted down without interference or comment. The terms were registered exactly in the order of mention and later the frequency of mentioning across informants and the salience was calculated. This part of the research therefore resulted in an extensive gross list of WFPs, CPs and CRs (valuing the neglect) gathered and consumed during period of food stress, but also provided details on (absolute and relative) frequency of mention of individual species and cultural salience. Using field guides of Ethiopian flora, local farmer knowledge, key informants/local experts and their experience and expertise, the principal investigator and the field assistant/translator converted the list of foodstuffs (including crop residues) into a list of edible parts and the plants they originated from, based on local names. The gross list was complemented with additional list of WFPs, CPs, and CRs obtained through key informant interviews and own observations made in crop fields, residential areas and other sites. Then this comprehensive list was used for the next phase of the research, i.e. the focus group discussions. Moreover, the gross list was used to compile a list of scientific names of WFPs and crops.

### Key informant interviews

Seven key informants, all grown up and living in the study area, including three academically educated agriculturalists and four farmers who were considered knowledgeable of the farming systems and culture, were identified with the help of local government offices and local community leaders based on their level of knowledge in the food security situation of the area and interviewed by the first author who had repeatedly met the key informants to also clarify unclear issues as needed. In fact two of the key informants (experts) had previously participated in and served as field assistant for other botanical surveys carried out in the district and hence were very instrumental in generating and clarifying most of the points posed by the principal researcher.

### Focus group discussions

Subsequently, three independent focus groups (each composed of 8-10 illiterate people, who showed good knowledge during the individual interviews and were prepared to share their knowledge) were requested to categorize the WFPs, CPs, and CRs from the gross list obtained through interviewing the households into different categories based on the food situation during which they were gathered and consumed. The participants in the focus group discussions were mainly identified based on their good knowledge about WFPs observed during the free listing exercises and those who had listed only few items were excluded from the focus group discussions. Knowledge on WFPs, CPs, and CRs is often gender specific due to gender differences in division of labor [29,30]. In mixed focus groups women often are less candid and they often behave differently compared to single-sex focus groups. Therefore the three focus groups differed: group 1 consisted of only women, in group 2 there were only men, whereas group 3 consisted of both women and men. The purpose of organizing the three focus groups, however, was not to find out differences across gender per se but rather to build and validate consensus among the focus groups on the items listed in each category as an endorsement or confirmation. These focus groups divided the WFPs, CPs, and CRs into the following three categories: WFP, CPs, and CRs gathered and consumed in normal situations (*Hira*; green light), WFPs, CPs, and CRs gathered and consumed when available food stock is alarmingly low (locally termed *Sharshara*; amber light), and WFPs, CPs, and CRs gathered and consumed when people face severe food shortage (locally termed *Lawota kokoka*; red light). These sequential circumstances are synonymous to normal, warning, and emergency times, respectively, with regard to food security. From the gross list created by the free-listing, key informant interviews and own observations, the researcher and the translator verbally called out the name of each plant one-by-one (in no particular order) during the focus group sessions and requested the focus group members to classify the plant according to the three categories. Each focus group then classified the plants into one of the three specific categories and the names were written down on a flip chart using green, amber and red colors to represent normal, caution and disaster situations, respectively. During the three independent exercises, the participants in each focus group argued and reached consensus in allocating each plant or plant product to one of the three categories. When plants were put in the same category by at least two focus groups, there was agreement and thus endorsement.

### Plant species

Plant parts and plant species identified by the interviewees were further elaborated by the principal researcher based on a field guide of wild food plants and flora of Ethiopia, in close collaboration with knowledgeable key informants and interviewees (for local names) mastering the local language. The fourth author, who is a botanist at Addis Ababa University (Ethiopia) and has done several botanical studies in the study area, also played a key role in identifying plant species, either directly in the field or based on specimens collected.

Thirty five voucher specimens were collected from different microenvironments in the main rainy season (mid-February to mid-June, 2009) by the principle researcher with the assistance of knowledgeable informants (male and female) and the translator. The researcher pressed the specimens, identified the species and reconfirmed the species names at Addis Ababa University Herbarium using Ethiopian flora volumes with the help of the fourth author of this paper. The voucher specimens were deposited at the Herbarium for future references.

### Validation of data

The data presented in this study refer to the situation in the midlands of Konso. However, we surmise that in each agro-ecological setting it will be possible to identify amber and red species that can be used as key-indicators of impending food stress or of the severity of the stress. We also surmise that the more similar (in terms of agro-ecological and socio-cultural settings) certain environments are, the more likely it is to have the same set of plant species signaling for the amber and red situation. We therefore compare our findings with those from previous studies in southern Ethiopia in which the first author was involved [5,6] and with data from a PhD study carried out in Hamar (southern Ethiopia) and defended at Addis Ababa University [8].

## Results

### Results of the free-listing

Tables 1 and 2 illustrate that, based on the type and parts of wild food plants or crops eaten, there are seven types of foods consumed. These types are locally termed 1. *Unta* (for cereals like sorghum, maize, barley, wheat and finger millet); 2. *Kharsha* (pulses like haricot bean, pigeon pea, cow pea and other beans); 3. *Tinisha* (root and tuber crops such as Irish potato, sweet potato, taro, yam and cassava and the different types of *pakanna*); 4. *Mietaa* (raw eaten tubers or roots from plants like *kullayya*, etc.); 5. *Midha* (leafy vegetables eaten cooked like cabbage, *moringa* and tomato leaves); 6. *Luqata* (fruits eaten raw such as *liya*, *helteta*, *hinkikata*, etc.); and 7. *Hawudheta* (plants and stems like sugarcane, sorghum stalk and maize stalk).

**Table 1:** Wild food plants (WFPs) gathered and consumed under the traffic light metaphor in Konso, Ethiopia

**WFPs parts gathered and consumed under the red household food situation (mainly based on consumption by adults)**				
**No.**	**WFPs**	**Scientific name**	**Part(s)**	**Remarks**
1	*Dhahanata*	*Cucumis dipsaceus* Spach.	Leaves	
2	*Hakala koyra*	*Erucastrum pachypodum* (Chiov.) Jonsell	Leaves	Wild cabbage
3	*Hanqarara*	*Senna singueana* (Delile) Lock	Leaves	
4	*Liltota*	*Arisaema flavum* (Forssk.) Schott	Leaves	Thorny
5	*Teyla*	*Brassica napus* L.	Leaves	If eaten alone frequently
6	*Dhashandhashata*	Plant species not confirmed	Fruits	Fruits are with very little flesh
7	*Ikayteta*	Plant species not confirmed	Fruits	Consumed like juice
8	*Kahelta*	*Psydrax schimperiana* A. Rich. Bridson	Fruits	Tiny fruits and the plant is rare
9	*Manqoraya*	Rhus sp. (Anacardiaceae)	Fruits	Paralyzes teeth when consumed
10	*Heriya*	*Lagenaria siceraria* (Mol.) Standl.	Seeds	Eaten roasted
11	*Achacha* (wild/false millet)	Elusine sp. (Poaceae)	Grains	Never eaten alone even in bad times
12	*Yetota* (wild sorghum)	*Sorghum arundinaceum* (Desv.) Stapf	Grains	Shatters very easily; kept for bad times
13	*Karsata*	*Dobera glabra* (Forssk.) Juss. ex Poir.	Kernels	Rock-hard kernels, long hour boiling
14	*Hakata*	Plant species not confirmed	Roots	Adults only in bad times
15	*Heqayata*	*Cyperus bulbosus Vahl*	Roots	
16	*Kullayya sakkarra*	Vatovaea sp. (Fabaceae)	Roots	
17	*Kuritata*	*Dorstenia barnimiana* Schweinf.	Roots	
18	*Napa arpa*	Plant species not confirmed	Roots	
19	*Tombolasha*	Tacazzea sp. (Asclepiadaceae)	Roots	Fresh or cooked; watery
20	*Pakanna* (*Qachama*)	*Amorphophallus gomboczianus *Pic. Serm	Tubers (mature)	Very irritating taste (never eaten alone)
21			Tubers (immature)	
22			Tubers (fresh)	Needs adequate desiccation before processing it to food
23	*Inkutayata*	*Boletus sp.* (Boletaceae)	Shoots	Rare; some poisonous, prudently collected
24		*Agaricus campestris* L.	Shoots	
25	*Maraeta (Qitqita)*	Portulaca sp. (Portulacaceae)	Whole plant	Frequent consumption will induce diarrhea
**WFPs parts gathered and consumed under the amber household food situation**				
26	*Honona*	*Justice flava* (Vahl) Vahl	Leaves	Only available during wet times
27	*Ifayata*	*Ocimum sanctum* L.	Leaves/shoot	Spice, but in food shortage times eaten as a cabbage
28	*Isatta*	*Kedrostis pseudogijef* (Gilg) C. Jeffrey	Leaves	Normally used as medicine, but as food in bad time
29	*Katata*	*Commiphora kataf* (Forssk.) Engl.	Leaves	Boiled and drunk like coffee
30	*Qannatata*	*Pouzolzia parasitica* Schweinf.	Leaves	Boiled; tasteless
31	*Rasuta*	*Amaranthus graecizans* L*.*	Leaves	Frequent intake without other foods induces diarrhea
32	*Chalanchalota/Hepata*	*Flueggea leucopyrus* Willd.	Fruits	It is always eaten fresh/raw
33	*Chaqalesa*	*Grewia erythraea* Schweinf.	Fruits	Adults only in bad times
34		*Grewia ferruginea* Hochst. ex A. Rich.	Fruits	
35	*Gnarqeta*	Plant species not confirmed	Fruits	
36	*Hakala*	Brassica sp. (Cruciferae)	Fruits	
37	*Hotarta*	Plant species not confirmed	Fruits	
38	*Kabutayta*	*Rhus natalensis* Bernh. ex Krauss	Fruits (dried)	Kernel is hard and always discarded
39	*Kombolta/Babalota/ Qulqualeta*	*Opuntia ficus-indica* (L.) Mill.	Fruits	Thorns are tiniest and very dangerous if entering into eyes
40	*Liya*	*Ficus platyphylla* Del.	Fruits	
41	*Luqata sigmama*	*Cadaba farinosa* Forssk.	Fruits	Roots have medicinal value for animal diseases; if eaten by adults it shows food stress
42	*Maqayta*	*Euclea racemosa* subsp. *schimperi* (A. DC.) F. White	Fruits	
43		*Euclea divinorum* Hiern	Fruits	
44	*Marka*	Plant species not confirmed	Fruits	
45	*Mudhukanta*	*Vangueria madagascariensis* J.F. Gmel.	Fruits	
46	*Pa-ata (Ye bereha lomi)*	*Sclerocarya birrea* (A. Rich.) Hochst.	Fruits	
47	*Porporissa*	*Ehretia cymosa* Thonn.	Fruits	Tiny, less tasty
48	*Punita purkaya*	*Lantana trifolia* L.	Fruits	“False coffee”; fruits are small
49	*Qignfirta*	*Combretum aculeatum* Vent.	Fruits	Available in lowlands
50	*Timpliqisha*	*Vangueria apiculata* K. Schum.	Fruits	
51	*Tinayta*	*Ficus ingens* (Miq.) Miq.	Fruits	
52	*Korahiteta*	Plant species not confirmed	Roots	
53	*Oritata*	Lannea sp. (Anacardiaceae)	Roots	
54	*Qahata*	*Commiphora terebinthina* Vollesen	Roots	Medicinal value in delivery
55			Bark	Smells bad
**WFPs gathered and consumed even under the green household food situation**				
56	*Akaketa*	Plant species not confirmed	Leaves	
57	*Arjohela*	Plant species not confirmed	Leaves	
58	*Hankalta*	*Balanites aegyptiaca* (L.) Delile	Leaves/ tendril	Succulent, bitter
59	*Hankolayta*	*Launaea intybacea* (Jacq.) Beauv.	Leaves	Available during times of food shortage/wet times
60		*L. taraxacifolia* (Willd.) Amin	Leaves	
61	*Kasha*	Plant species not confirmed	Leaves	
62	*Kepata*	Plant species not confirmed	Leaves	
63	*Khayla*	*Leptadenia hastata* (Pers.) Decne	Leaves	Invasive weed, like cabbage
64	*Kugnarata*	*Lablab purpureus *L.	Leaves	
65	*Masara*	Vatovaea sp. (Papilionaceae)	Leaves	
66	*Moqorqorsa/Moroqroqsa*	*Oxygonum sinuatum* (Hochst. & Steud. ex Meisn.) Dammer	Leaves	Available during times of food shortage/wet times
67	*Okalata*	Dolichos sp. (Fabaceae)	Leaves	
68	*Oloqiloqota*	*Corchorus trilocularis* L.	Leaves	Only available during periods of food shortage (wet times)
69	*Pasa*	*Amaranthus hybridus* L.	Leaves	Watery leaves
70			Seeds	
71	*Poshanata*	Plant species not confirmed	Leaves	
72			Roots	
73	*Qananta*	*Berchemia discolor* (Klotzsch) Hemsl.	Leaves	Sweet, sticky
74			Fruits	Good taste
75	*Qaqaha/Qahaqaha*	*Solanum nigrum* L.	Leaves	Cooked
76			Berries	
77	*Qaqula*	*Adenia ellenbeckii* Harms	Leaves	Available during rainy season; protected
78	*Rasuta*	*Amaranthus graecizans* L*.*	Leaves	Eaten mixed with other food items
79		*Amaranthus dubius* Mart. ex Thell.	Leaves	Only available during periods of food shortage in wet times
80	*Shabalata*	Plant species not confirmed	Leaves	
81	*Teyla*	*Brassica napus* L.	Leaves	Eaten mixed with other food items
82	*Torchata*	*Celosia argentea* L.	Leaves	Delicious; occurs in March and April
83	*Torqeta*	*Celosia trigyna* L.	Leaves	
84		*Digera muricata* (L.) Mart.	Leaves	
85	*Agamta*	*Carissa spinarum* L.	Fruits	Fruits temporarily desensitize teeth after consumption
86	*Cheqerta*	*Ficus thonningii* Blume	Fruits	Small fruits
87	*Dhahayta*	*Grewia bicolor* Juss.	Fruits	
88		*Grewia velutina* (Forssk.) Lam.	Fruits	
89	*Halota*	Plant species not confirmed	Fruits	Sweet, usually available in lowlands
90	*Hankalta*	*Balanites aegyptiaca* (L.) Del.	Fruits	Fresh/dried; good storability
91	*Hashashayata*	Cordia sp. (Boraginaceae)	Fruits	
92	*Heleta*	*Ficus sur* Forssk.	Fruits	
93	*Hillteta*	*Ficus sycomorus* L.	Fruits	
94	*Hinkikata (Enkoy)*	*Ximenia caffra* Sond.	Fruits	Eaten fresh/raw
95	*Kopta*	*Ziziphus mucronata* Willd.	Fruits	Pulp is very acidic and not liked in normal times
96	*Kumanata*	*Physalis peruviana* L.	Fruits	
97	*Kutata*	*Balanites rotundifolia* (Tiegh.) Blatt.	Fruits	Sweet, mostly occurs in lowlands, availability is seasonal
98	*Lamtta*	*Blyttia fruticulosum* (Decne.) D.V. Field	Fruits	Difference in use only in age
99	*Madherta*	*Cordia sinensis* Lam.	Fruits	Occurs in lowlands
100	*Mordhoha*	Balanites sp. (Balanitaceae)	Fruits	
101	*Ogomteta*	*Grewia villosa* Willd.	Fruits	Usually available in lowlands; sweet
102	*Otayta*	*Cordia africana* Lam.	Fruits	Pounded and mixed with salt
103	*Poketa*	Plant species not confirmed	Fruits	
104	*Qaraqawola*	Plant species not confirmed	Fruits	
105	*Qehakorba*	Plant species not confirmed	Fruits	
106	*Qochata*	*Grewia ferruginea* Hochst. ex A. Rich.	Fruits	Also has medicinal value
107		*Grewia bicolor* Juss.	Fruits	
108	*Qopissa (Poqsa)*	*Grewia villosa* Willd.	Fruits	Sweet
109	*Rokoota*	*Tamarindus indica* L.	Fruits	Slightly sour/bitter
110	*Toleqota*	*Cordia monoica* Roxb.	Fruits	Sticky
111	*Qawureta*	*Sterculia africana* (Lour.) Fiori	Seeds (Beans)	Eaten in two forms: drank like coffee, or eaten roasted like beans
112	*Alge-heluta*	Plant species not confirmed	Roots	
113	*Kullayya*	*Vatovaea pseudolablab* (Harms) J. B. Gillet	Roots/Tubers	Planted/protected on terraces
114	*Mechata*	Plant species not confirmed	Roots	
115	*Pakanna (Ateta)*	Amorphophallus sp.	Mature tubers	
116	*Poshanposha*	Plant species not confirmed	Roots	Eaten fresh
117	*Saitata*	Plant species not confirmed	Roots	
118	*Checheha*	*Acacia hockii* De Wild	Bark (inner)	
119	*Lahadho*	Plant species not confirmed	Whole plant	
120	*Parapaqa*	*Pachycymbium laticoronum* (MG Gilbert) MG Gilbert	Whole plant	Whole plant is eaten except the fine roots

Within a scale plants species are grouped based on parts eaten and subsequently listed in alphabetical order of the trivial name.

**Table 2:** Crop parts (A for red), crop parts (B for amber) and crop residues (C for red), and recycled stuff (D for red) gathered and consumed under the traffic light metaphor in Konso, Ethiopia

**A) Crop parts gathered and consumed under the red light metaphor**				
**No.**	**Crop**	***Scientific name***	**Part(s)**	**Remarks**
1	Coffee (*Punita*)	*Coffea arabica* L.	Leaves (*hasha*)	If drunk alone frequently; normally used mixed with other food items
2	*Hidhana*	Dioscorea sp. *(Dioscoreaceae)*	Leaves	
3	*Hohe/Aylenata*	Semi-wild bean type	Leaves	
4			Husks	
5	Irish potato (Tinisha)	*Solanum tuberosum* L.	Leaves	Cooked and eaten
6	Khat *(Timahata)*	*Catha edulis* (Vahl) Forssk. ex Endl.	Leaves (including old ones)	When ingested instead of being chewed or sold
7	Moringa *(Shalaqayta/Midha)*	*Moringa stenopetala* (Bak.) Cuf.	Leaves	Normally consumed mixed with other food items (green light), but it gives diarrhea if eaten alone frequently (red light)
8	Pepper (*Parpara*)	*Capsicum frutescens* L.	Leaves	
9	Pumpkin *(Potata)*	*Cucurbita pepo* L.	Leaves	Normally only fruits are eaten
10	Sweet potato (Dhedhero)	*Ipomoea batatas* (L.) Lam.	Leaves	Cooked and eaten
11	Taro (*Kansata*)	*Colocasia esculenta* (L.) Schott.	Leaves	
12			Stem (*Longa*)	
13	Tomato (*Gnagna*)	*Solanum lycopersicum* L.	Leaves	Eaten as cabbage
14	Banana (Muzita)	*Musa paradisiacal* L.	Fruit (immature)	Eaten boiled
15			Corm	
16	Papaya (Papaye)	*Carica papaya* L.	Fruit (immature)	Eaten boiled
**B) Crop parts gathered and consumed under the amber light metaphor**				
17	Cassava (*Moko*)	*Manihot esculenta* Crantz	Leaves	Boiled to remove
18			Root sheath	harmful/side effects of its consumption
19	*Shuqata*	Vigna sp. (Fabaceae)	Leaves	
20	Haricot beans	Phaseolus sp. (Fabaceae)	Seeds/beans (defective)/*tocha*)	If eaten mixed with other food items it shows amber light
21	Enset/false banana *(Dhubana)*	*Ensete ventricosum* (Welw.) Cheesman	Corm (immature)	Eaten boiled
**C) Crop residues/left-over consumed under the red light metaphor**				
1	Haricot beans	Phaseolus sp. (Fabaceae)	Seed/beans (defective)/*Tocha*	Eaten alone
2			Pods (empty)/*Qofalla*	
3	Barley	*Hordeum vulgare* L.	Husks/bran	Locally called *tata porta*
4	Maize	*Zea mays* L.	Husks	Boiled with coffee leaves and eaten
5			Tassels *(Fita)*	
6	Finger millet	*Eleusine coracana* (L.) Gaertn.	Bran (*Ukkasha*)	Normally, used as labor payment/gift for destitute; when started to be consumed by better off households then the light is turned on red
7	Sorghum	*Sorghum bicolor* (L.) Moench	Bran (*Ukkasha*)	
8	*Dhohotota*	*Cajanus cajan* (L.) Millsp.	Pods (empty)	Boiled/cooked
9	*Lukluka*	Assorted	Powdery left-overs	Boiled/cooked
10	Cotton (*kulma putota)*	*Gossypium hirsutum* L.	Seeds	Boiled for drink
**D) Recycled stuff consumed under the red light metaphor**				
11	*Tata*	Different types (mainly sorghum or maize)	Local drink residue	Recycled for local beer/gin

Within a section of the table, crop species are grouped based on crop part eaten and subsequently listed in alphabetical order of the trivial name.

Based on the oral free-listings, key informants interviews and field observations a list of 152 unconventional food stuffs from WFPs, crop parts, crop residues and recycled stuff was obtained of which 119 were from the free-listing exercise and 33 came from key informants or own observations. All items consisted of foodstuffs that did not consist of the normal edible crop parts that are harvested and commonly used as food: 120 based on wild food plants (Table 1), 21 based on crop parts, 10 based on crop residues, and one recycled stuff (Table 2). This list contained over 130 different plant types or folk plant names (over 110 wild food plants and more than 20 names of crops or crop categories). The WFPs included more than 100 scientific plant names, although 23 have not been identified yet (Table 1). It should be noted that some plant species can provide different foodstuffs (edible parts) and that some foodstuffs identified by folk names can be provided by different plant species. Informants mentioned another 10 wild food plant species for which we could not obtain enough reliable information to include them in Table 1.

Among the wild food plants, listed in Table 1, the diversity of the plant parts eaten is large, but fruits, leaves, root and tubers are dominant. The semi-managed wild food plant *pakanna*, producing edible tubers, was the most frequently mentioned (by more than 90% of the interviewees). It was also by far the most abundant wild food plant in the study area, mainly in agro-environments. The Konso communities use it as an insurance against hunger because of its good storability in the ground and because it is self-proliferating. The name *pakanna* is colloquially used for at least three types or species: *ateta, panshala/rometa* and *qachama* (in order of decreasing preference and increasing pungency). *Pakanna* is normally consumed in association with other (staple) food items. Each of the three types differ in their ecological and management requirements, color and size of the tubers, size and shape of the leaves, time of maturity and availability, taste and methods of preparation. *Ateta* is an early maturing, short cycle plant and its tubers are white. *Panshala* is medium early while *qachama* is late. *Qachama* is the most pungent and most irritating one and hence needs the greatest preparation effort to make it into edible foodstuff. In the traffic light metaphor, the stage of harvesting (mature or immature), consumption (mixed or alone), frequency and intensity could be interpreted accordingly. Consumption of *qachama* without any additional food items clearly indicates that the amber light is on and if the family in question is repeatedly consuming it alone then the household is in the red zone.

The crop parts eaten in times of food stress include the three dominant ones, leaves, immature fruits, and corm, but also husks, stem, seed/beans, and roots/root sheaths (Table 2). The crop residues eaten in times of food stress, listed in Table 2, are defective seeds and beans, pods, husks, bran, and tassels. Recycled stuff was also sometimes eaten (*tata*). These foodstuffs have poor taste and are often poorly digestible for humans.

### Classification on the basis of focus group discussions

Household food stock availability and/or depletion in the metaphor of a traffic light: green, amber and red indicates levels of food availability from normal, alert and emergency status, respectively. When the food situation can be classified as green, people do gather wild food plants, which are mostly tasty and readily available, accessible, and less cumbersome in processing [5,6,8]. They do so to diversify their diets. In the amber situation, i.e. when the food stocks at the household level is alarmingly low, more wild food plants but also some crop residues are gathered and consumed, but only those that are not harmful to the health condition of the household members. They do so to increase the volume of intake. However, this is the transition phase to the red phase, the phase in which the food reserves and other assets are completely depleted and people try to stay alive by consuming food sources that might directly undermine their health condition.

The results of the classification by the three focus groups (women, men and mixed group) were used to summarize the extent to which WFPs, CPs, and CRs were used in normal times or at different levels of food shortage (Tables 1 and 2).

Table 1 also illustrates that some wild food plants are eaten under all circumstances. The gathering and consumption of some wild food plants as such cannot be taken as an indicator of food shortage in the Konso context. The best cases in point are gathering and consumption of the leaves of *khayla* (*Leptadenia hastata* (Pers.) Decne), a creeping evergreen liana which is one of the top most preferred green vegetables in Konso and Hamar districts [6,8], or the leaves of *torchata* (*Celosia argentea* L.) and *torqeta* (*C. trigyna* L.). These food items are considered delicious and be eaten any time, but the plants are not considered as crops. Despite its wide abundance and availability mostly at road sides and on waste-lands and its desirable features, *khayla*, for instance, is not promoted in agro-ecosystems fields because of its creeping, invasive and suppressive nature, among other things.

Seventeen crops were listed for which various crop parts (seven types) were used as food under amber (4) and red (13) light conditions. Seven crops were listed for which various residues, usually not consumed by humans (including broken seeds and beans, pods, husks and bran, and tassels) were used as emergency food (10 foodstuffs; Table 2). All of these crop residues were classified as red light condition foods. Leaves of coffee (*hasha*) and leaves of *moringa* are special cases: they are consumed as a side dish but when they are repeatedly eaten alone this is an indication of (emerging) food shortage. When a household starts consuming a drink of coffee leaf (*hasha*) as soup alone i.e. without additional foods with it, this is an indication of the amber light condition and if this is happening repeatedly, then this family is in the red zone. The same applies to *Moringa stenopetala* (Bak.) Cuf. *Moringa stenopetala* is always consumed as part of the meal but usually not as the sole food as this will result in diarrhea. Thus when a family starts consuming *Moringa stenopetala* alone as a meal, the family is in the amber zone and when this happens repeatedly it is getting into the red zone. So through close and continuous monitoring of the food situations in the study area, for example in Konso, it is possible to locally detect the food availability trend and know whether the food situation is deteriorating or not, at least based on the Konso context. This monitoring should start well before the two hunger seasons (mid-September to October/November and April/May to mid-June [25-27]), when different WFPs are more abundant due to the *Belg* and *Meher* rains [5,6,8] and the people are gathering and consuming. It should be done specifically by the development agents living within the rural community.

### Relations with food stress severity

On the basis of Tables 1 and 2, we roughly but objectively classified foodstuffs with a marking or signaling value into 9 different categories based on some features to better summarize and understand the various reasons why different foodstuffs obtained from wild food plants or crop residues have signaling value (Table 3). Table 3 gives a descriptive classification of WFPs, CPs, and CRs, provides species names for the different classes and provides some relevant remarks.

**Table 3:** Categorization of wild food plants (WFPs) based on some features such as unavailability, taste, processing difficulties, and health risk at the time of gathering/consumption/or after intake

	**Categories**	**WFPs in each category**	**Remarks**
1	Plants which are rare or difficult to find nearby.	*Dobera glabra, Cordia sinensis, Acacia hockii,* and *Balanites rotundifolia*	*Dobera glabra* is found only in remote lowland areas.
2	Plants consumed normally mixed with other food items, but under severe food shortage eaten alone.	*Moringa stenopetala, Pachycymbium laticoronum*, *Adenia ellenbekii, Coffea arabica, Amaranthus dubius, Brassica napus,Corchorus trilocularis,* Vigna sp., Portulaca sp.	
3	Those normally eaten only by children but eaten by adults in bad times.	Many of the WFPs fall in this category.	Consumption of these WFPs by adults points to a change in the availability of food and indicates incoming or prevalence of food shortage.
4	Plants, immature plant parts with less nutritious, irritating taste.	*Amorphophallus gomboczianus*, *manqoraya *(Rhus sp.), *Tamarindus indica*	Immature tubers of *Amorphophallus gomboczianus* (harvested in the first year when normally it ripens in 2-3 years). Has poor palatability and also has irritating taste. *Manqoraya* fruits temporarily desensitize/paralyze teeth after consumption. *Tamarindus indica* fruits have sour taste.
5	Plants with low yield of edible parts/products, relatively poor taste.	*Kullayya sakkarra*, *Amaranthus hybridu*s, *Ehretia cymosa*, *Lantana trifolia, dhashandhashata* (species not identified), *Psydrax schimperiana*	*Kullayya sakkarra* tubers are diminutive*,* not palatable but are nonetheless consumed under critical food shortage. *Amaranthus hybridus* tiny seeds with bitter taste.
6	Products which are extremely hard to process or to cook to food; sometimes also sour.	*Dobera glabra, Grewia ferruginea,G*. *bicolor*	Gathering and consumption of *Dobera glabra* (kernel/fruit) clearly indicates that the household consuming it has entered the worst stage of food shortage/starvation as the kernel is rock-hard to be boiled. It could also lead to diarrhea and stomach ache. *Grewia ferruginea* and *G*. *bicolor*: tough to process because its cover cannot easily be removed. *Amorphophallus gomboczianus* tubers are hard to process to food.
7	Watery weeds or parts normally consumed only after adequate desiccation to avoid diarrhea.	*Pachycymbium laticoronum*, and leaf of Amaranthus sp. which would normally require desiccation before consumption.	Frequent consumption of these stuffs is deemed unhealthy by the Konso community.
8	Plants or plant parts which are dangerous to handle.	*Opuntia ficus-indica, Arisaema flavum*	Fruits of *Opuntia ficus-indica* bear the finest/tiniest spines which will blind in case of entering into the eyes. *Arisaema flavum* -thorny and difficult to collect leaves.
9	Plants which are health threatening after intake, deemed poisonous and if consumed are perceived as fatal.	Some types/species of mushroom (Boletus sp.) are poisonous and cannot be consumed without careful management/cooking.	Only collected by adults, not by children to avoid child health risks.

Note that some plants can appear in more than one category.

In properly interpreting their signal value, aspects of availability and abundance, role in the diet, consumption pattern, taste and nutritious value, the need for processing, and health risks before or after ingestion need to be taken into account.

However, it is necessary to stress that during the interviews and the focus group discussions it became clear that people are also showing pro-active behavior. Especially the women of Konso, being the most responsible for daily food preparation and availability, used measures available to reinforce their household food supply. Women already store or keep crop residues (valuing the neglect) or gathered wild food plants in the kitchen, under the house roof, in the backyard and in small containers after drying them, even during times of relative food abundance. For example, even when production is abundant, women preserve crop residues, by-products of local beer or local liquor to be recycled for food (locally known as *tata*), etc. for two reasons: either for food in times of great need or for cattle feed if the situations remain normal. Husks of maize, pods of pulse crops (*qofalla)* are deliberately kept under the roof, in small bags and in the cases of root/tuber weeds in the ground/soils as a reserve for bad times.

### Quantitative analysis of frequency of mention of species in the green, amber and red categories

Results of the interviews are further detailed in Table 4. Since in some cases species identification was based on colloquial names, the data set includes 119 species, with a summed number of mentions of 4236 (Table 4). Interviewees were capable of listing many different species. The mean numbers of amber and red light species were high, despite the fact that overall more green light species were mentioned by the 220 households.

**Table 4:** Total number of mentionings, total number of species listed by all households and average number of species mentioned per household (all species, and for green, amber or red species, separately) based on a free-listing exercise among 220 households in Konso

	**All species of wild food plants or crop parts and residues**^**1**^	**Green species**	**Amber species**	**Red species**
Total number of mentionings among 220 households	4236	723	1466	2047
Total number of species listed	119	56	20	43
Average number of species mentioned per household	19.3	3.29	6.66	9.30

^1^ Note that the numbers are based on the free listing only and therefore slightly deviate from the sums of the total numbers from Tables 1 and 2.

Based on their frequency of mention and cultural salience the most prominent or salient species in the study area were *pakanna*, *maraeta*, *karsata*, *hinkikata*, *qaqula*, *pasa*, *qochata*, *kullayya*, *parapaqa*, *khayla*, *hankalta*, *otayta* and *rasuta* in that decreasing order of salience. *Pakanna* and *maraeta* are colloquial names and include a number of species sharing the same name. Among all these items, *karsata* (*Dobera glabra* (Forssk.) Juss. ex Poir.) is found out to be the single most salient and most frequently mentioned item despite its unfavorable characteristics as food; it has poor palatability for human beings, induces abdominal pain and diarrhea and requires painful processing to become “edible”.

### Validation of the consistency of the traffic metaphor

Amber and red species can be used as key-indicators of impending food stress or of the severity of the stress. Which species can perform this signaling function depends on plant ecological aspects, the agro-ecology of the area and the socio-cultural attitude of the people towards plants. For example, in Konso, evaluating which type of *pakanna* is used and how it is eaten will already provide a gauge of the level of food stress. The same is true for how maize tassels or chaff and bran of grain crops are used. Based on previous studies from literature [5,6,8], we can verify whether our classification of WFPs could also work in other agro-ecologies in southern Ethiopia. Although the plants we have identified as having a large signaling value are not always present in all agro-ecologies and data sets differed in type and method of data collection, there is a remarkable resemblance for some of the key species with a strong signaling character. The consumption of *karsata* (*Dobera glabra* (Forssk.) Juss. ex Poir.) by households in both agro-ecologies (lowlands and midlands) of Konso district and in Hamar district (have 75 species and associated ethnobotanical knowledge in common [8]) indicates that households in these three contexts are already in the red zone. Its boiling requires long hours [5,6,8] (according to Konso women informants it takes about half a day to boil it and make it ready as “food”), consumes lots of fuel wood and water which are scarce in the study area. Similarly, the consumption of *hasha* and *moringa* alone in the two agro-ecologies in Konso indicates an amber or red phase, while consumption of the same stuff in repeated frequency indicates red phase in both agro-ecologies; consumption of *qachama* similarly indicates severe food shortage in both agro-ecologies. The consumption of *khayla* (*Leptadenia hastata* (Pers.) Decne) in the three contexts indicates households are in their normal food availability phase/green metaphor. The consumption of *ateta*, *khayla*, *kullayya*, *torchata*, *hasha* and *moringa* mixed with other food items all indicate a normal food situation (green).

## Discussion

### Amber and red species

We developed the traffic light metaphor as an early warning system for food stress, based on gathering and consumption of various WFPs, CPs, or CRs. Based on the comprehensive list of WFPs, CPs, and CRs and plant identification, more than 100 species of wild food plants and 24 crop species could be identified as being consumed in times of food stress and detailed information on them could be substantiated, although for a considerable number identification still needs to be verified (Tables 1 and 2). Based on the information provided by the informants and the focus discussion groups we could categorize the amber and red species into 9 different groups based on a set of descriptive criteria and we could list the species for each of these groups (Table 3) to further specify the traffic light metaphor.

In his ethnobotanical study conducted in Hamar and Xonso (Konso) districts of Ethiopia where 143 WFPs for Hamar and 138 for Konso were recorded Getachew [8] reported 11 and 16 WFPs (*Dobera glabra, Portulaca quadrifida* and *Launaea intybacea* in common) consumed under chronic food shortage/famine (red light) and, 18 and 12 WFPs (with at least five species in common) gathered and consumed during food scarcity (amber light) in Hamar and Konso communities, respectively.

Farmers in the midlands of Konso were capable of listing many species of WFPs, CPs, and CRs gathered and consumed that could serve as indicators for their food security. In general a total of 20 amber species, 43 red species and 56 green species were mentioned (Table 4). Some informants were capable of listing more than 40 species, some red species were listed by almost all informants, whereas on average informants mentioned about 16 species in the categories of amber and red species combined (Table 4). It is obvious from this information that Konso men and women are very knowledgeable on what nature can offer them when food is scarce.

### Relations of gathering with severity of food stress

In cognizance of the recurrent drought-induced crop failure and hence food shortage (for example from 1996-1999 [5,6]), farmers in Konso (southern Ethiopia) have developed and used indigenous, ethno-botanical and ethno-ecological knowledge to cope with food stress. In food shortage periods as the stock of food becomes depleted, and especially when severe food stress is faced (usually from April to mid-June) households start relying more on wild food plants of a different kind, even those having undesirable features, or start consuming unconventional foodstuff and *recycled* by-products or residue of local beer, in addition to crop left-overs. Different types of *pakanna* tubers (*Amorphophallus gomboczianus*) are collected in this period and are particularly relied upon. When the food situation worsens specific foodstuffs start to become part of the gathering basket. Eating unattractive and sometimes risky or health-threatening products is a practice most likely developed through and based on multi-generational experience of the indigenous communities.

### Red species and health

It is well known that species categorized as red can be harmful to human health and are therefore reserved for use as an extreme coping strategy. In the Konso and Hamar districts (Ethiopia), 31% (n = 138) and 38% (n = 143) wild foodstuffs mentioned, respectively, by one or more informants, have some sort of adverse effects on human health, mainly abdominal pain and diarrhea [8]. It is these types or categories of food items which are consumed when the traffic light is red and such consumption patterns should provide an alert for humanitarian partners monitoring the seasonal food situation and assessing the livelihoods of subsistence rural households of Konso. Consumption of such WFPs could be used as an accurate indicator of severe food stress. For example, consumption of *karsata* (*Dobera glabra* (Forssk.) Juss. ex Poir.) kernels is the classical indicator of severe food shortage in Konso and Hamar districts [6,8]. This is because the kernels are rock-hard and hardly cooked and when consumed lead to diarrhea, stomach aches and other health complaints by the users.

### The value of the traffic light metaphor

In early phases of a food crisis, people will concentrate on the wild and famine foods that are nutritious and certainly not health-threatening, but in the most severe stages of famine people will even start to gather and eat famine foods which will be health-threatening.

We have schematized this gradual change into a traffic light metaphor, based on the type, time and intensity of gathering and consumption of WFPs, CPs, and CRs. Three distinctive types of information are incorporated. *First,* there are gathering and consumption patterns of wild food plants that do not signal an abnormal situation. *Second,* at the other extreme, the gathering and consumption of undesirable and health-threatening wild plants and the consumption of crop residues (never been considered as stress indicator before, but valued and considered as a classical indicator of food stress in our study) indicate severe food stress and we identified quite a few of those plants and residues (see also [5,6,10,31,32]). *Third*, there is an intermediate or transition phase. In that phase the food stock is starting to deplete but is not yet exhausted and hence there is a change from normal to abnormal. This transition phase could signal a worsening situation and is also characterized by gathering and consumption of specific crop residues and wild food plants or by a change in dominance of these food sources in the diet.

This analysis of the phases and intensity in use of wild food plants and of the use of crop parts and crop residues as food stress indicator differs from the conventional thinking in the area of disaster studies of assuming that any collection and consumption of WFPs is an indication of food stress. The mere collection of wild food plants is not a signal of severe food shortage in Konso. Instead, gathering and consumption of specific wild food plants (including other products indicated) is an accurate indicator of emerging food insecurity and when this situation progresses gathering and consumption of specific plants can be further used as indicators in the assessment of the severity of food stress. These indicators provide a cheap, locally available, and easy to understand means to monitor and assess the food situation, provided area and socio-cultural specific data (like for other food security indicators including, for instance, market data for food grains and animal prices) on type and time of collection of the specific WFPs are reliable and relevant. With a detailed analysis of the gathering and consumption mainly by adults, one can assess how alarming the food situation of households in the area is as part of an early-warning system. The metaphor of the traffic light will help to make such a system more accountable, effective and accurate, complementing the national conventional early-warning systems for future food crises. This new type of stress indicator could further be used for identifying and targeting the most vulnerable groups and food insecure people or households [4,33,34] for relief responses and/or for recovery programs meant to save lives or to restore the livelihoods of the households.

On a reasonably narrow range of agro-ecological and socio-cultural conditions the red species are the same. However, for other communities, the amber species might differ and in agro-ecologies with very different physical conditions, the occurrence and abundance of red species will differ as well.

### How to use the traffic light system?

Items 56-120 of Table 1 are always gathered and consumed and have no discriminative power. Items 1-25 in Table 1, items 1-16 and all items in the sections C and D of Table 2 have little value for the objective of early warning as the food crisis is already there, but have value when the (changes in) frequency and intensity of consumption of these items are monitored in detail to assess the severity of the food stress. Items 26-55 in Table 1 and items 17-21 in Table 2 indicate people are in the amber phase and detailed information on what they gather here can provide an early warning signal. So local people could be regularly interviewed by asking how often they have consumed certain plants. Questions should be like: Have you eaten during the last few days any of the following WFPs, CPs or CRs and if yes, how often? If a certain number is recorded they can be in the amber zone, if the number is high and the species mentioned are of very poor quality or even health threatening then the conclusion can be that these people are in the red zone and need immediate external support. Observations of the specifics of the WFPs gathered and CPs and CRS consumed by the community members by the local development personnel living within the community is an important way of gathering similar information on the local food availability situations.

Using the traffic light metaphor provides a more precise signal than the crude indication of number or quantity of WFPs, CPs, and CRs gathered and consumed. Although our system will require more detailed research, focusing on key species in the amber and red categories could already provide enough information to draw reliable conclusions for an evaluator.

### Can the traffic light metaphor be generalized?

The introduction of the traffic light metaphor as a food (un)availability indicator in rural areas of southern Ethiopia sharpens food situation monitoring information and improves the quality and reliability of early-warning information based on the contextualized coping behaviors of households. Its applicability in other areas of the country with the similar context, preceded with similar studies, will improve the national early-warning system of the country in general and the study areas in particular. The applicability of the metaphor is strongly associated with the breaking down of the WFPs/foodstuffs occurring in the specific study area(s) into different phases of food (un)availability and will have a corrective measure to the generic use of WFPs as an indicator of food stress in the national food security early-warning system. Our approach avoids the use of the conventional assumption that collection and consumption of any WFPs is an indication of (severe) food stress which by implication also undermines the nutritional role of WFPs (eaten as green leaves, flowers, fruit, seeds, pods, shoots, roots/tubers and so on) as a normal part of the cuisine. Its use and applicability is further maximized in rural areas where each Farmer Association (FA; the lowest administrative unit in rural areas of Ethiopia) has got a number of its own development agents/personnel living and working with and carrying out monitoring of household food availability as routine tasks in their respective areas and making seasonal food security information/data on food (un)availability ready for the larger national monitoring system and for international humanitarian partners.

Our approach is most likely also valid in other chronically food insecure regions of Ethiopia where the use of wild food plants is both a part of the normal diet and part of the coping strategy in the context of food shortage. Context-specific behaviors tend to be indicators of more severe coping and thus help to identify severely food insecure households [4]. We therefore recommend analogous studies in other parts of Ethiopia where similar recurrent food stress occurs and where communities intensively use WFPs, CPs, and CRs to cope with the food stress, but it will not be necessary to repeat similar studies for each Farmers’ Association, *woreda* (equivalent to district) or agro-ecological zone.

### Calibration against other coping strategies indices

Humanitarian agencies and emergency partners use the Coping Strategies Index (CSI) developed as a context-specific indicator in measuring food insecurity which counts up and weights coping behaviors at the individual household level [4]. In the Konso district and the Hamar district (both are distinct in ethnicity, culture, language and socio-economic settings, with the Hamar district being west of Konso), 96% (n = 335) and 95% (n = 315) of the informants, respectively, experienced incidence of recurrent food shortage in their areas at least once in their lifetime [27]. In these study areas, focus groups reported reducing meal portion size, reducing number of meals per day, eating less-preferred foods, food aid, WFPs gathering and consumption, selling of commodities/assets, support from others, other income sources/labor and hunting as important coping mechanisms in periods of food stress times where wild food plants consumption was ranked/considered as the first (in Konso) and the second (in Hamar) most important coping mechanism [6,27]. There is a core set of behaviors that are relied upon across a variety of contexts, in more or less the same order of frequency. Each type of behavior is perceived as having relatively the same severity across study areas and cross-contextual comparison using coping strategies indices becomes possible [4]. Eating wild foods, consumption of seed stock and going entire days without food demarcate serious food shortage and should be recognized as indicators of severe food stress [cf. 4].

In comparison with the recent developments in food security and famine early-warning scales (IPC, FEWSNET, etc.) which involve more levels of scale (for instance, five-level by FAO/FSAU [3]) the current traffic light metaphor is an easier tool to be used at the grass-root level. It also permits and encourages community level participation in the process where literally visual observations are used in early-warning systems for food stress.

Nevertheless, it is fair to state that it will not be necessary to replace existing scales. Therefore it is wise to gradually scale up the use of the traffic light metaphor in close association with the continued use of existing scales in order to let it prove its value. Calibration needs to be done at a wide scale in order to avoid mistakes by oversight of local specifics [4]. We are convinced that in areas where gathering wild food plants under food stress is intense and where indigenous knowledge plays an important role in the pro-active management of these resources, this concept will be applicable, so not only in southern Ethiopia but also elsewhere in Africa and in the hunger-stricken world at large.

### Final remarks and conclusions

Our study supports the conclusion of Maxwell et al. [4] that local-specific behaviors should continue to be the focus of index construction with the presumption that more context specific indices (having more individual behaviors with inclusion of more severe ones) provides a better chance of accurately capturing context specific food insecurity and thus, the traffic light metaphor is a meaningful and a plausible food (in) security indicator.

We argue that the consumption of some specific wild food plants involves risks because they can be harmful to human health or even lethal [Desalegn (19) noted lethal cases of people in Wello (Ethiopia) due to eating some type of WFPs] when eaten without extended processing. Gathering and consumption of these wild food plants is viewed as a clear indication of extreme severity of the food stress. Therefore, we strongly recommend that the use of human health threatening foodstuffs be included in the CSI in the enumeration of consumption-related coping strategies for monitoring food security status.

We have complemented the conventional seasonal food security assessments used by humanitarian partners (Government, NGOs and UN agencies) by providing an easy, cheap approach to scale food stress encountered by subsistence farmers. We recommend analogous studies in other parts of Ethiopia where recurrent food stress also occurs and where communities intensively use crop residues and wild food plants to cope with food stress. The consumption of *karsata* (*Dobera glabra* (Forssk.) Juss. ex Poir.) is envisaged to be the best indicator of critical food stress (red metaphor) in Konso and also in Hamar district [5,6,8].

## Abbreviations

CP: Crop part; CR: Crop residue; CSI: Coping Strategies Index; FA: Farmers’ Association; WFP: Wild food plants.

## Competing interests

The authors declare they have no competing interests.

## Authors’ contributions

DLO, PCS and LLP conceptualised and designed the research. DLO identified the study topic and the study area based on his local food security and ethnobotanical experience in the study area through his extensive food security monitoring in the country. EK supported the study in providing technical guidance in the collection, identification and nomenclature of plant specimens and facilitated depositing of the specimens in the National Herbarium of Addis Ababa University. KK provided a field guide and served as a translator and mediator between the local community, local officials and the study team, thus helping the study team in the daily field activities. DLO, PCS, EK and KK contributed to data collection and data verification. All authors contributed to the writing of the manuscript.

## Authors’ information

Dechassa Lemessa Ocho is an agronomist by profession and has worked as food security researcher and expert for more than 20 years, including more than 15 years with government, international non-governmental organizations and the UN OCHA (Office for the Coordination of Humanitarian Affairs), both at national and international level, in Africa, Asia and the USA and has done extensive research on food security and the occurrence of wild food plants in different parts of Ethiopia, including Konso. He worked with the UN in New York, USA, as a Food Security Officer for the Horn of Africa, including Ethiopia and is currently working for South Sudan UN OCHA.

Paul C Struik (Professor of Crop Physiology, specialized in seed systems and agrobiodiversity at Wageningen University, Wageningen, the Netherlands) and LLP (Professor of Anthropology specialized in ethnobiology and food studies at Oregon State University, Corvallis, Oregon, USA) jointly supervise research on wild food plants in Africa and Asia, PCS focusing on methodological and ecological aspects and LLP focusing on anthropological aspects and socio-cultural methodological aspects.

Ensermu Kelbesa (Professor of Biology at Addis Ababa University, Addis Ababa, Ethiopia and Curator of the National Herbarium in Ethiopia) has specific expertise in botany and occurrence of wild food plants in Ethiopia and especially in the Konso region. He has conducted and supervised several ethnobotanical studies in Ethiopia.

Koshana Kolo is an academically trained agriculturalist originating from the study community and is currently doing his M.Sc. studies in Ethiopia on WFPs in Konso as the current study helped him initiate his interest in the same subject while he was supporting our field work and he wanted to make use of his extensive knowledge of the local situations in Konso.

## References

[B1] Famine scales http://en.wikipedia.org/wiki/Famine_scales

[B2] Howe P Devereux S Famine intensity and magnitude scales: A proposal for an instrumental definition of famine Disasters 2004 28 4 353 372 10.1111/j.0361-3666.2004.00263.x 15569378

[B3] Food and Agriculture Organization/FSAU Integrated Food Security and Humanitarian Phase Classification. Technical Manual Version 1 2006 11 Nairobi, Kenya: FAO/FSAU Technical Series IV

[B4] Maxwell D Caldwell R Langworthy M Measuring food security: Can an indicator based on localized coping behaviors be used to compare across contexts? Food Policy 2008 33 533 540 10.1016/j.foodpol.2008.02.004

[B5] Guinand Y Dechassa L Wild Food Plants in Southern Ethiopia: Reflections on the role of wild food plants and famine foods at a time of drought (unpublished) 2000 Addis Ababa, Ethiopia: United Nations Emergency Unit for Ethiopia

[B6] Guinand Y Dechassa L A Practical Field Guide to Wild Food Plants in Ethiopia: General description, edible parts, preparation methods and palatability 2001 Addis Ababa, Ethiopia: UNDP-Emergency Unit for Ethiopia 110

[B7] Youngblood D Identification and quantification of edible plant goods in the Upper (Nama) Kardoo, South Africa. Econ Bot 2004 58 1 S43 S65 10.1663/0013-0001(2004)58[S43:IAQOEP]2.0.CO;2

[B8] Getachew A Edible wild and semi-wild plants of Hamar and Xonso (South Ethiopia) with emphasis on their ethnobotany and nutritional composition of selected species 2009 Addis Ababa University, Addis Ababa, Ethiopia: PhD thesis

[B9] Shackleton SE Dzerefos CM Shackleton CM Mathabela FR Use and trading of wild edible herbs in the Central Lowland Savannah Region, South Africa Econ Bot 1998 52 3 251 259 10.1007/BF02862142

[B10] Getachew A Kelbessa U Dawit D Ethnobotanical study of edible wild plants in some selected districts of Ethiopia Human Ecol 2005 33 1 83 117 10.1007/s10745-005-1656-0

[B11] Young H Food scarcity and Famine: Assessment and response Oxfam Practical Health Guide No. 7 1992 London: Oxfam Publications

[B12] Turner NJ Davis A When everything was scarce: the role of plants as famine foods in northwestern North America J Ethnobiol 1993 13 171 201

[B13] Desalegn R Local coping mechanisms in Wello, Ethiopia J Ethiopian Studies 1998 28 23 51

[B14] Dechassa L Food Situation Assessment Report for Konso Special District, SNNPR. UN Emergency Unit for Ethiopia, Field Mission Report; 5-15 October 1999, Addis Ababa, Ethiopia 1999 5 15

[B15] Disaster Prevention and Preparedness Agency (DPPA) National Food Security Situation Assessment Reports 1984 Addis Ababa, Ethiopia

[B16] Corbett J Famine and household coping strategies World Development 1988 16 9 1099 1112 10.1016/0305-750X(88)90112-X

[B17] Nyok MA Foustino C Food security and the role of indigenous wild food plant in South Sudan *The potential of Indigenous Wild Food Plants* 2001 Kenya: Proceedings from the Workshop held in Diani, Kenya 22 26

[B18] Muchomba E Sharp B *Southern Sudan Livelihood Profiles. A guide for humanitarian and development planning* Second edition, May 2007

[B19] Desalegn R Famine and survival strategies: A case study from Northeast Ethiopia. Food and Famine Monograph series No. 1. Addis Ababa: Institute of Development Research, Addis Ababa University 1991

[B20] Zemede A Mesfin T Prospects for sustainable use and development of wild food plants in Ethiopia Econ Bot 2001 55 1 47 62 10.1007/BF02864545

[B21] Gemedo DT Maass BL Isselstein J Plant biodiversity and ethnobotany of Borana pastorals in Southern Oromia, Ethiopia Econ Bot 2005 59 1 43 65 10.1663/0013-0001(2005)059[0043:PBAEOB]2.0.CO;2

[B22] Kebu B Fassil K Ethnobotanical study of wild food plants in Derashe and Kucha Districts, South Ethiopia J Ethnobiol & Ethnomed 2006 2 2 53 10.1186/1746-4269-2-2 17184523 PMC1769355

[B23] Haile Y Yewhalaw D Teketay D Ethnomedicinal plant knowledge and practice of the Oromo ethnic group in Southwestern Ethiopia J Ethnobiol & Ethnomed 2008 4 11 10.1186/1746-4269-4-11 18445249 PMC2390512

[B24] Population Census of Ethiopia Central Statistical Authority of Ethiopia 2007 Ethiopia: Addis Ababa

[B25] FARM Africa Report of the study on pre-emergency employment in Konso Special Woreda, October, 2001 2001 Ethiopia: Addis Ababa 60

[B26] Famine Early Warning System (FEWS NET) Southern Nations Nationalities and People’s Region (SNNPR) Livelihoods Profiles, Regional Overview 2005 FEWS NET and DPPA/B, December

[B27] Famine Early Warning System (FEWS NET) Ethiopia Food Security Outlook, October 2009 to March 2010 2009 USAID Funded Project’s Biannual Magazine: USAID Funded Project’s Biannual Magazine

[B28] Sutrop U List task and a cognitive salience index. Field Methods 2001 13 263 276 10.1177/1525822X0101300303

[B29] Madge C Collected food and domestic knowledge in the Gambia, West Africa Geographical J 1994 160 3 280 294 10.2307/3059610

[B30] Howard PL (ed.) Women and plants 2003 New York, USA: MacMillan: Gender relations in biodiversity management and conservation 320

[B31] Amare G The role of wild plants in the native diet in Ethiopia Agro Ecosystems 1974 1 45 56

[B32] Abebe D Kelbessa U Debela A Ambaye C Dejene A Survey of poisonous plants in Ethiopia Ethiopian J Health Devel 2001 15 3 209 221

[B33] Coates J Frongillo E Rogers B Webb P Wilde P Houser R Commonalities in the experience of household food insecurity across cultures: what are measures missing? Supplement to the J Nutr 2006 5 136 1438 1448 10.1093/jn/136.5.1438S 16614441

[B34] Coates J Swindale A Bilinsky P Household food insecurity Access Scale (HFIAS) for Measurement of Household Food Access: Indicator Guide (v.2) 2006 Washington, DC, USA: Food and Nutrition Technical Assistance Project, Academy for Educational Development

